# The effect of mindfulness-based counseling on the childbirth experience of primiparous women: a randomized controlled clinical trial

**DOI:** 10.1186/s12884-023-05607-4

**Published:** 2023-04-21

**Authors:** Bahare Sharegi Oskoui, Esmat Mehrabi, Roghaiyeh Nourizadeh, Khalil Esmaeilpour

**Affiliations:** 1 Student Research Committee, Midwifery Department, Tabriz University of Medical Sciences, Tabriz, Iran; 2 Midwifery Department, Faculty of Nursing and Midwifery, Tabriz University of Medical Sciences, Tabriz, Iran; 3 Faculty of Education and Psychology, University of Tabriz, Tabriz, Iran

**Keywords:** Mindfulness, Child birth experience, Child birth pain

## Abstract

**Introduction:**

Unpleasant childbirth experience is considered as one of the important factors for cesarean preference. Limited studies have been investigated the impact of psycho-based interventions on the childbirth experience and the most effective counseling approach to promote a pleasant childbirth experience has not been clearly identified.

**Objective:**

The present study aimed to evaluate the impact of mindfulness-based counseling on the childbirth experience of primiparous women.

**Methods and materials:**

This randomized controlled clinical trial was conducted on 64 primiparous with gestational age of 32 to 34 weeks referred to the perinatology clinic of Al-Zahra and Taleghani educational-treatment hospital, affiliated to Tabriz University of Medical sciences, Iran. Participants were randomly assigned into the intervention and control groups. The intervention group received eight mindfulness-based counseling sessions. The intensity of childbirth pain with VAS (Visual Analog Scale) in the active phase of labor at 8 cm dilatation and the childbirth experience questionnaire were completed by interview after childbirth. Independent t-test and ANCOVA were used to compare the outcomes between the two groups.

**Results:**

After controlling the effect of confounding variables, the mean score of childbirth experience in the intervention group was significantly higher than that in control group [Mean Difference (MD): 1.79, 95% CI: 2.52 to 1.07, *P* < 0.01]. The mean score of labor pain in the intervention group was significantly lower than that in the control group after controlling the effect of baseline score and confounding variables [MD: -2.21, 95% CL: -3.35 to -1.10, *P* < 0.001].

**Conclusion:**

The findings indicated that providing mindfulness-based counseling improves the childbirth experience and reduces labor pain during childbirth. However, further randomized clinical trials are needed before making a definitive conclusion.

**Trial registration:**

Iranian Registry of Clinical Trials (IRCT): IRCT20171007036615N9.

Date of registration: 16/03/2022, 25/12/1400.

## Background

Achieving a high proportion of a safe and attractive normal vaginal birth (NVB) is a challenge for obstetrical care providers [1]. If a nulliparous woman achieves a NVB, her chance for a future NVB increases [2–4] and if she is not satisfied with her childbirth experience, the risk for a Caesarean section (CS) increases due to woman’s choice for elective caesarean [5, 6].

Nowadays, approximately 140 million births occur worldwide annually [7] and most of these births do not have any known risks for the mother and the fetus. However, various interventions (such as oxytocin induction, epidural analgesia) are employed during vaginal delivery to start, accelerate, regularize, and terminate pregnancy. While these medical interventions during the childbirth process can have negative sideffects, for example, although the growing use of Oxytocin during labor and delivery might be associated with beneficial effects, its use may lead to an increased risk of adverse outcomes [8, 9].

These unfavorable effects may lead to an unpleasant childbirth experience and women’ preference for CS, which is obviously accompanied by many complications for the mother and the fetus in the short- and long-term [10, 11]. In addition, the childbirth experience is recognized as an influencing factor on the physical and mental state of the mother during the postpartum period [12–15].

The childbirth experience is complex and the effectiveness of pain relief is considered as only one of many dimensions that may affect a laboring woman’s overall experience with the labor and childbirth process. In addition to the degree of pain relief, any modality chosen may also impact a woman’s (and her partner’s) sense of focus, control, well-being, satisfaction, and feelings of support [16]. Further, it should be considered that maternal expectations, the support of caregivers and health-care providers, and perceived degree of control or autonomy over the process are more important determinants of satisfaction with the childbirth experience compared to pain relief provided [17, 18].

Further, traumatic childbirth experience can have many negative effects, including poor mother-baby bond, unwillingness to breastfeed [19], posttraumatic stress disorder (PTSD) [20], and poor quality of life [21]. Although these negative experiences can fade over time, they may persist in women’s memory for up to five years [18].

Scientific evidence denoted that maternity care and interventions focused on reducing labor pain can be effective in creating a pleasant childbirth experience [16]. Although the existed pharmaceutical methods are used to reduce the intensity of labor pain, none of pharmaceutical analgesia are without complications, reducing the desire to use them [22]. For example, a greater demand for epidural as pharmaceutical analgesia is associated with the widespread use of oxytocin for both labor induction and augmentation. The association with a higher incidence of CS is still debated with conflicting results between large descriptive studies and RCTs which can result in unpleasant childbirth experience [23–27].

Among the non-pharmacological methods for labor pain management and other chronic pain, mindfulness-based counseling method has been used in some studies [22, 28]. However, the review studies conducted on the effectiveness of mindfulness-based counselling approach in pain management indicated the study gap in this field to achieve definitive results [29–31]. World Health Organization (WHO) emphasized conducting studies on the impact of psychological aspects on the childbirth experience and the reduction of elective cesarean sections to decrease unnecessary cesarean Sects. [32].

Considering that the importance of creating a pleasant childbirth experience has been stressed by WHO, finding solutions based on scientific evidence to improve this outcome is an important step in promoting Spontaneous Vaginal Delivery and reducing maternal death as one of the sustainable development goals. Given the lack of studies in the field of the impact of counseling and various interventions on the childbirth experience to identify the impact of non-pharmacological and psycho-based intervention methods, the present study aimed to assess the impact of mindfulness-based counseling on understanding the labor experience and pain.

## Methods

Participants were randomly assigned into the intervention and control groups. The intervention group received eight mindfulness-based counselling sessions. The intensity of labor pain in the active phase of labor at 8 cm dilatation and the childbirth experience questionnaire were completed by interview after childbirth. Independent t-test and ANCOVA were used to compare the outcomes between the two groups.

### Study design and participants

This two-arm randomized controlled clinical trial was conducted on 64 primiparous women referred to the perinatology clinic of Taleghani educational-treatment hospital, affiliated to Tabriz University of Medical sciences, Iran, from March 2022 to June 2022. The primigravida women with a gestational age of 32—34 weeks and a cephalic singleton pregnancy and without mental disabilities or deafness who intended to give birth in the afore-mentioned hospitals participated in the study. The exclusion criteria included a history of mental disorders before pregnancy based on the health proflie, abnormal volume of amniotic fluid, having any surgery on the uterus, non-reactive NST, fetal abnormality, high-risk pregnancy, including hypertension, diabetes, placenta previa, and cardiovascular and other chronic diseases, bleeding in the third trimester, placental abruption, fetal growth disorder, pregnancy with the assisted reproductive technologies, the desire to receive other pharmacological and non-pharmacological analgesia methods, unplanned pregnancy, any contraindications for vaginal delivery, including Cephalopelvic disproportion (CPD).

This study was part of a master’s thesis, investigating the effect of mindfulness-based counseling on the experience, support, and control of childbirth as a primary outcome and labor pain and fear as a secondary outcome.

## Sample size

The sample size was calculated based on the mean score of childbirth experience and according to the study of Ghanbari-Homayi et al. (2019) [33]. Considering M_1_ = 2.70 (mean score of childbirth experience), M_2_ = 3.37 (assuming a 25% increase due to the intervention), SD_1_ = SD_2_ = 0.7, α = 0.05, and Power = 80%, sample size was estimated 26 subjects in each group. Finally, the sample size was considered 32 in each group based on the variables of support and control in childbirth and regarding 20% attrition.

### Sampling and random assignment

This trial was conducted on primiparous women at 32–34 weeks of gestation referred to the specialized perinatology clinic of Al-Zahra and Taleghani hospitals in Tabriz. The sampling was done after obtaining permission from the Ethics Committee of the Deputy of Research and Technology of Tabriz University of Medical Sciences (IR.TBZMED.REC.1400.1121) and Tabriz Faculty of Medical Sciences and registering on the Iranian Registry of Clinical Trials (IRCT) site (IRCT code: 20171007036615N9). The researcher (the first author, who was a senior student of midwifery counseling and had the certificate of mindfulness-based counselling course) attended the specialized perinatology clinic of the afore-mentioned hospitals and examined the inclusion criteria. The eligible women were requested to attend a face-to-face session in one of the clinic’s rooms and the objectives and method of the study were fully explained. Then, the written informed consent form was obtained from the women willing to participate in the study and socio-demographic and obstetric characteristics questionnaire was completed. In our hospital, epidural analgesia services are not provided for Spontaneous Vaginal Delivery, due to the inadequacy of skilled and expert personnel in this field. Other pharmacological and non-pharmacological methods are offered to the mother according to the doctor’s prescription, and childbirth care is mainly doctor-oriented and it is not common to use pharmacological and non-pharmacological pain relief methods.

The participants were assigned into the intervention (mindfulness-based counselling) and control groups with a ratio of 1:1 by blocked randomization using Random Allocation Software (RAS) with a block size of 4 and 6. Blocking was done by a non-involved person in sampling and data analysis. The type of allocation was written on a piece of paper and put in opaque envelopes numbered consecutively for allocation concealment. The envelopes were opened in the presence of the mother by a non-involved person in the sampling process (clinic manager of hospital).

### Intervention

The intervention group (mindfulness receiving group) received eight counseling sessions in a combination of four online and four face-to-face sessions for 45 min once a week. The homework requested from the mothers was checked by the researcher and feedback was given to the mother. Similar to the intervention group, the control group received routine antenatal care education in a combination of four online and four face-to-face sessions (Table 1).

**Table 1:** The content of consultation

Content	Session
Introducing, explaining mindfulness as a way of life, practicing mindful eating, giving feedback and discussing on mindful eating and body practice Homework: 45 min of meditation treatment, paying daily attention to active and continuous care through bathing, eating a meal mindfully during the week	One
Yoga, discussing on the interaction between mindful activities, familiarizing with homework, discussing about mindful attitude, introducing sitting meditation, sitting meditation guide (10 min), giving feedback and discussing on sitting meditation Homework: 45 min of meditation treatment, ten minutes of breathing mindfully, paying attention to various ongoing daily routines to record the experience of a pleasant event during the day	Two
Yoga, sitting meditation (15 min), listening and seeing practices (5 min), discussing on the mindfulness attitude (non-judgmental), classifying the mindfulness, including introducing the dialogue of the mindfulness as an exercise, meditation guide, and one minute breath walking Homework: Continuing and practicing breathing on Sundays, Tuesdays, Thursdays, practicing movements consciously on Saturdays, Mondays, and Wednesdays. Daily recording of pleasant experiences, three minutes breathing for three times in a day	Three
Yoga, sitting meditation, including mindfulness toward breathing, body, sounds, and thoughts, discussing on the mindful attitude, including acceptance and relinquish, addressing problematic emotions Three minutes breathing—offering exercises to use when we experience difficult emotions Walking with mindfulness	Four
Yoga, sitting meditation, including mindfulness of breathing, body, sounds, and thoughts, introducing problematic thoughts and memories, 3-min breathing exercises Homework: Creating relaxation and mindfulness, including normal 3-min breathing, reviewing three times a day, and 3-min of patterned breathing when we experience unpleasant feelings	Five
Yoga, sitting meditation, including mindfulness of thoughts, breathing exercises for anger management, mindfulness and communication, and mountain and lake meditation Homework: Practicing for 40-min every day—working with different variations of the three main exercises, including 3-min normal breathing, 3-min patterned breathing when unpleasant feelings occur, and reflecting and working on a plan to prevent personal relapses	Six
Yoga, sitting meditation, including mindfulness of breath, body, sounds, and thoughts, mindfulness and compassion, and meditation Homework: Choosing all of the different forms of exercises, giving a common pattern to the person so that they can continue them after the program termination, and normal and practiced breathing	Seven
Yoga, sitting meditation, conclusion, and discussion on how to integrate mindfulness practices into our lifestyle	Eight

### Data collection tools

The data were collected using the socio-demographic and obstetric characteristics questionnaire, Childbirth Experience Questionnaire version 2 (CEQ2), and Visual Analogue Scale (VAS) for labor pain. The socio-demographic and obstetric characteristics questionnaire was complete before intervention in both group.The intensity of labor pain was measured using Visual Analog Scale (VAS) in the active phase of labor at 8 cm dilatation and the childbirth experience questionnaire was completed by interview after childbirth.

### Socio-demographic and obstetric characteristics questionnaire

The questionnaire included the variables of age, education, occupation, family income, medical interventions during labor, such as prescribing drugs inducing uterine contractions, etc.

Content and face validity were used to determine the validity of the socio-demographic and obstetrics characteristics questionnaire. The questionnaire was given to the faculty members. Based on the feedback received, the corrections were made on the tools after collecting their opinions.

### Childbirth Experience Questionnaire version2) CEQ2)

This questionnaire with 22-item was developed by Dencker et al. (2010) to measures four main domains of the childbirth experience, including own capacity, professional support, perceived safety, and participation. The revised version of the instrument with 23-item is scored using a 4-point Likert scale ranging from 4 (totally agree), 3 (mostly agree), 2 (mostly disagree), to 1 (totally disagree) [34]. Iranian version of this questionnaire is a valid and reliable tool for measuring sleep quality. The reliability of Iranian version of this instrument with Cronbach’s alpha was reported to be 0.93 [33].

### Visual Analogue Scale (VAS)

In this scale, the participants marked the severity of their pain on a 10-cm ruler with one end stating “no pain” and the other end “worst possible pain”. VAS was reported to be more sensitive and reliable in pain assessment compared to the other single-dimensional scales [35]. Therefore, it was used to determine the severity of pain and the effectiveness of the intervention in this study.

### Data analysis

The data were analyzed using SPSS_26_ software. First, the normality of the distribution of the studied quantitative outcomes, including pain intensity and childbirth experience, was investigated by the groups using the Kolmogrov-Smirnov test. Considering the normality of the data distribution, ANCOVA test was used to compare the mean score of pain severity in the intervention and control groups after the intervention by controlling the effect of the baseline score and participation in postpartum preparation classes as confounding factors.

## Results

**Fig. 1:**
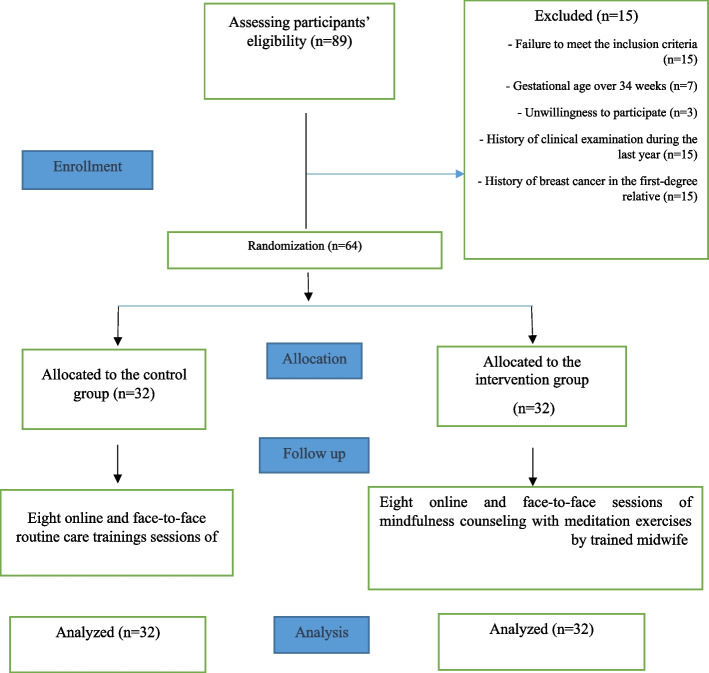
Flowchart of the study

Sampling started in February 2022 and continued until May 2022. The researcher evaluated 89 pregnant women with gestational age of 32–34 weeks, of whom 64 met the inclusion criteria. Further, 32 women were allocated randomly to the counseling group, and they participated in 8 counseling sessions. There was no drop in the study and 64 women were retested and the data were analyzed after the intervention (Fig. 1).

The mean (SD) age of women was 24.34 (4.33) and 23.34 (5.48) years in the intervention and control groups, respectively. More than two third of participants in two groups received oxytocin for childbirth induction and approximately half of them in both groups received pethidine, hyoscine, and promethazine as labor pain relief during the active fase of labor (*P* > 0.05) (Table 2).

**Table 2:** The socio-demographic and obstetrics characteristics of participants

Variables		Intervention group (*n* = 32) Number (%)	Control group (*n* = 32) Number (%)	*P*-value
Age *		24.34 (4.33)	23.34 (5.48)	0.42*
Spouse’s age^*^		28.88 (4.36)	29.59 (5.22)	0.52*
Level of education	Secondary school	21 (65.62)	18 (56.25)	0.71**
	High school / diploma	9 (28.12)	12 (37.50)	
	Bachelor's degree and higher	2 (6.25	2 (6.25)	
Occupation	Housekeeper	28 (87.50)	29 (90.62)	0.43**
	Employed outdoor	4(12.50)	2 (6.25)	
	Employed indoor	0 (0.0)	1 (3.12)	
Family income level	Enough	0 (0.0)	1 (3.12)	0.46**
	Somewhat enough	16 (50.0)	16 (50.0)	
	Not enough	16 (50.0)	15 (46.88)	
Wanted pregnancy	Yes	32 (100.0)	31 (96.96)	0.31**
	No	0 (0.00)	1 (3.03)	
Labor induction with oxytocin	Yes	0 (0.00)	1 (3.03)	0.44**
	No	2 (6.25)	3 (9.38)	
Pharmacological labor pain relief (pethidine, hyoscine and promethazine)	Yes	14 (43.75)	15 (46.87)	0.18**
	No	18 (56.25)	17 (53.12)	

Independent T-test *

Chi square test **

Based on the ANCOVA test and by adjusting the effect of confounding factors (participation in childbirth preparation class), the total mean score of childbirth experience in the intervention group was significantly higher than that in the control group [MD: 1.79, 95% CI: 2.52 to 1.07, *P* = 0.01]. Further, the mean score of the domain of personal capacity, professional support, perceived safety, and participation in the intervention group was significantly higher than that the control group, respectively [MD: 1.77, 95% CI: 2.65 to 0.93, *P* = 0.001], [MD: 1.91, 95% CI: 2.70 to 1.12, *P* = 0.01] [MD: 1.57, 95% CI: 2.18 to 0.90, *P* = 0.001], and [MD: 1.77, 95% CI: 2.61 to 0.95, *P* = 0.001] (Table 3).

**Table 3:** The comparison of the childbirth experience score in the intervention and control groups after the intervention

Variable	Intervention group Mean (SD)	Control group Mean (SD	MD^a^ 95% CI	*P*
Personal capacity	3.62 (0.7)	1.85 (0.5)	1.77 (2.65 to 0.93)	0.001*
Professional support	3.61 (0.9)	1.71 (0.3)	1.91 (2.70 to 1.12)	0.001*
Perceived safety	3.86 (0.3)	2.29 (0.5)	1.57 (0.90 to 2.18)	0.001*
Participation	3.53 (0.9)	1.76 (0.7)	1.77 (2.61 to 0.95)	0.001*
Total score	3.79 (0.7)	2.00 (0.8)	1.79 (2.52 to 1.07)	0.001*

^a^MD with 95% CI

ANCOVA * by adjusting the confounding effect (Oxytocin induction, postpartum hemorrhage, participating in childbirth preparation classes)

^b^The acquired score range is between 1—4

In addition, according to the ANCOVA test, the mean score of labor pain in the intervention group was significantly lower than that in the control group after the intervention [MD: 0.56, 95% CI: 0.37 to 1.50, *P* < 0.001] (Table 4).

**Table 4:** The comparison of labor pain score in intervention and control groups

Variable	Intervention group Mean (SD)	Control group Mean (SD)	MDa 95% CI	*P*
Labor pain (Acquired score range: 0–10				
Before 6 cm dilatation	7.72 (1.28)	7.09 (1.34)	-0.56 (0.37 to -01.50)	0.232*
After 6 cm dilatation	6.12 (1.28	8.33 (1.98)	-2.21 (3.35 to -1.10)	0.232*

^a^Mean Difference with 95% CI

ANCOVA * by adjusting the confounding effect (Baseline score and participating in childbirth preparation classes)

^b^The acquired score range is between 0—10

## Discussion

To the best of our knowledge, unfortunately, no study was found concerning the impact of mindfulness-based counseling and educational interventions on the childbirth experience. Therefore, the results of some relevant cross-sectional and qualitative studies and the studies examined the impact of care models on the childbirth experience were used to compare with the findings of the present study.

Ghanbari-Homaie et al. (2019) in a cross-sectional study investigated the effective factors in childbirth experience of 800 primiparous women during 1 to 4 months after giving birth. They reported that the psychological dimension influences the pleasant experience creation during pregnancy and childbirth and educational interventions during pregnancy should empower women to actively participate in childbirth care in order to create a pleasant childbirth experience [33]. These findings are consistent with the necessity of paying attention to the psychological dimension of childbirth and performing psycho-based interventions by improving the mother’s skills to participate in childbirth. In a randomized controlled clinical trial, McLachlan et al. (2015) examined the impact of midwifery-led care on women’s childbirth experiences in Melbourne, Australia and reprted that the mean score of childbirth experience in the intervention group was higher than that in the control group and mothers reported a positive experience of pain control (OR: 1.50, 95% CI: 1.22–1.84). Accordingly, mother’s participation in the delivery process and midwifery-led care compared to the standard care can improve childbirth experience of women [36], which is in line with the findings of the present study. Since interventions, delivery, and women’s support and guidance were performed by midwife and mothers used the mindfulness technique in the present study. Further, Hildingsson et al. (2019) in a randomized controlled clinical trial compared the effect of Internet-based cognitive behavioral therapy (CBT) and midwife counseling interventions on the childbirth experiences of women with fear of childbirth (FOC). The findings indicated that half of the participants reported the childbirth experience improvement and FOC reduction although there was no statistically significant difference between the groups in terms of the improvement of childbirth experience [37], The findings of afore-mentioned study are not in line with the results of the present study, due to the simultaneous holding of face-to-face and online sessions for pregnant mothers and the attendance of the researcher during childbirth to guide mothers to use mindfulness techniques during childbirth in the present study. While the interventions were performed only during pregnancy in the afore-mentioned study. Most of the studies were conducted on the education of participants after delivery or only in a limited period of time, while the present study provided counseling during pregnancy and researcher accompanied the mother and guided her to apply the mindfulness techniques during delivery. Further, the childbirth experience was measured using appropriate tools after giving birth. Finally, the findings of the present study and similar studies necessitate the application of mindfulness-based counseling programs to create a pleasant childbirth experience and empower mothers to actively participate in the delivery process during pregnancy. Therefore, the desire for elective cesarean section decreases and the desire for childbearing increases among mothers by creating a positive childbirth experience.

In addition, Gür et al. (2020) in a randomized controlled study investigated the effect of CBT techniques on labor pain of 273 pregnant women in Turkey. The findings demonstrated that the mean score of pain intensity significantly reduced in the intervention groups based on the visual analog scale (*p* < 0.05), [38], which is in line with the findings of the present study, illustrated the effect of psycho-based interventions on labor pain. Furthermore, Yuksel1 et al. (2017) in a randomized clinical trial examined the effect of breathing exercises on 250 pregnant women’s pain in the second stage of labor. Consistent with the findings of the present study, the results of the afore-mentioned study showed that breathing exercises with deep inhalation and exhalation were effective in reducing the labor pain perception of pregnant women [39]. Considering that breathing exercises are taught to mothers in the mindfulness technique, it is recommended to use breathing exercises during childbirth.

### Strengths and limitations

Some of the strengths of the present study included conducting an interventional study with random allocation, using the mother tongue of the participants in the counseling sessions, providing mindfulness-based counselling by a researcher who had a certificate of mindfulness skills with a high working experience in the delivery room, and using valid questionnaires, which their psychometric properties have already been evaluated in Iran. Given that the present study was conducted during the COVID-19 pandemic, participants were more inclined to hold online counseling sessions due to the fear of contracting the COVID-19, which may affect the results of the study. Also Covid-19 can affect maternal experience although non of participants reported no anxiety or worries concerning pandemic. Therefore, it is recommended to conduct a similar study with full face-to-face sessions after the end of the COVID-19 pandemic.

## Conclusion

For the first time, this study assessed the effect of psychological interventions on women’s childbirth experiences, which is a very complex issue.The results indicated that mindfulness-based counselling improves the childbirth experience and reduces the labor pain. Therefore, self-control methods during childbirth, can be learned through mindfulness-based counselling, during pregnancy. Accordingly, women can better manage the labor pain using mindfulness and their active participation in the process of giving birth creates a pleasant childbirth experience for them.

As study implications, the results of present study can help researchers design and present the different and effective care models, including psychological care and counsultations. Further, it is recommended that maternal health policy makers and planners make decisions on providing trained midwives with appropriate counseling skills to provide the necessary care for women during childbirth along with mindfulness-based education.

## Data Availability

The datasets used and/or analysed during the current study available from the corresponding author on reasonable request.
